# 

**Published:** 1880-01

**Authors:** 


					﻿r'QiiRG GENWM2FFSJ
Vol. xv.
January, 1880.
No. 4.
ThE
ISTDURY.
1 IMPONIw	vii ftp * JrJUJL&«
E .0 I T Q R ,
ELMmA.O.
				

